# Rapid Healing of Cutaneous Leishmaniasis by High-Frequency Electrocauterization and Hydrogel Wound Care with or without DAC N-055: A Randomized Controlled Phase IIa Trial in Kabul

**DOI:** 10.1371/journal.pntd.0002694

**Published:** 2014-02-13

**Authors:** Ahmad Fawad Jebran, Ulrike Schleicher, Reto Steiner, Pia Wentker, Farouq Mahfuz, Hans-Christian Stahl, Faquir Mohammad Amin, Christian Bogdan, Kurt-Wilhelm Stahl

**Affiliations:** 1 Mikrobiologisches Institut – Klinische Mikrobiologie, Immunologie und Hygiene, Friedrich-Alexander-Universität (FAU) Erlangen-Nürnberg and Universitätsklinikum Erlangen, Erlangen, Germany; 2 Leishmania Clinic, German Medical Service (NGO), Darwaze-e-Lahory, Kabul, Afghanistan; 3 Waisenmedizin e.V. Promoting Access to Care with Essential Medicine, Non-Profit Organization, Freiburg, Germany; National Institutes of Health, United States of America

## Abstract

**Background:**

Anthroponotic cutaneous leishmaniasis (CL) due to *Leishmania (L.) tropica* infection is a chronic, frequently disfiguring skin disease with limited therapeutic options. In endemic countries healing of ulcerative lesions is often delayed by bacterial and/or fungal infections. Here, we studied a novel therapeutic concept to prevent superinfections, accelerate wound closure, and improve the cosmetic outcome of ACL.

**Methodology/Principal Findings:**

From 2004 to 2008 we performed a two-armed, randomized, double-blinded, phase IIa trial in Kabul, Afghanistan, with patients suffering from *L. tropica* CL. The skin lesions were treated with bipolar high-frequency electrocauterization (EC) followed by daily moist-wound-treatment (MWT) with polyacrylate hydrogel with (group I) or without (group II) pharmaceutical sodium chlorite (DAC N-055). Patients below age 5, with facial lesions, pregnancy, or serious comorbidities were excluded. The primary, photodocumented outcome was the time needed for complete lesion epithelialization. Biopsies for parasitological and (immuno)histopathological analyses were taken prior to EC (1^st^), after wound closure (2^nd^) and after 6 months (3^rd^). The mean duration for complete wound closure was short and indifferent in group I (59 patients, 43.1 d) and II (54 patients, 42 d; *p* = 0.83). In patients with *Leishmania*-positive 2^nd^ biopsies DAC N-055 caused a more rapid wound epithelialization (37.2 d vs. 58.3 d; *p* = 0.08). Superinfections occurred in both groups at the same rate (8.8%). Except for one patient, reulcerations (10.2% in group I, 18.5% in group II; *p* = 0.158) were confined to cases with persistent high parasite loads after healing. *In vitro*, DAC N-055 showed a leishmanicidal effect on pro- and amastigotes.

**Conclusions/Significance:**

Compared to previous results with intralesional antimony injections, the EC plus MWT protocol led to more rapid wound closure. The tentatively lower rate of relapses and the acceleration of wound closure in a subgroup of patients with parasite persistence warrant future studies on the activity of DAC N-055.

**Trial Registration:**

ClinicalTrails.gov NCT00947362

## Author Summary

In many countries of the Middle East such as Afghanistan, cutaneous leishmaniasis is a highly prevalent, chronic and stigmatizing skin disease. Poor hygiene conditions frequently aggravate the lesions due to bacterial and fungal superinfections. Classical treatments with injections of pentavalent antimony are hampered by costs, side effects, resistance development, supply and manufactural quality problems. In the present study on Afghan patients with *Leishmania tropica*-induced skin lesions we evaluated the clinical effect of an initial removal of lesion tissue by electrocoagulation using a bipolar high-frequency electrosurgery instrument, followed by daily moist wound treatment with or without a preparation of pharmaceutical sodium chlorite (DAC N-055). DAC N-055 is a compound with anti-infective, immunomodulatory and tissue repair-promoting effects. Our analysis revealed that the carefully performed moist wound treatment led to a rapid healing of the wounds within an average period of 6 weeks, even in the absence of the sodium chlorite preparation. This is considerably faster than the time spans previously reported for local or systemic antimony treatment. We believe that the current standard for local care of chronic wounds should also be applied to *Leishmania* skin lesions. If combined with an initial single high-frequency electrocoagulation, it is a highly effective, inexpensive and well-tolerated treatment option for cutaneous leishmaniasis.

## Introduction

Human cutaneous leishmaniasis (CL) is a chronic infection caused by protozoan parasites of the genus *Leishmania* that are transmitted by sand fly vectors. In countries of the Near and Middle East such as Afghanistan *Leishmania (L.) major* and *L. tropica* are the principal parasite species that account for the development of the typical plaque-like or ulcerative skin lesions. The dermal lesions persist for months or even years, but eventually heal on their own (reviewed in [1]–[4]). However, the healing process can be complicated by superinfections, satellite lesions or a sporotrichoïd course of infection [1], [5]–[7] and commonly leads to disfiguring and socially stigmatizing scars [8]. From *L. major* mouse infections and from analyses of skin biopsies of patients suffering from *L. major* CL there is evidence that non-healing, ulcerative CL is linked to a diminished expression of genes involved in tissue remodeling [9] as well as to an upregulation of Fas ligand and tumor necrosis factor-related apoptosis-inducing ligand (TRAIL) [10].

Afghanistan is the country with the highest prevalence of CL with an estimated annual incidence of new active cases of more than 200,000 [11]. During a prevalence survey in Kabul, 2.7% of the population were found to have active disease in 2001 [12], which equals roughly 70,000 inhabitants and made Kabul the world capital of CL. The high prevalence of *Phlebotomus sergenti* and of *L. tropica* infections amongst humans accounts for the anthroponotic transmission mode of the disease in Kabul [12]–[14].

There is currently no standardized procedure for the management of CL following *L. tropica* or *L. major* infection. Whereas in the case of small or non-disfiguring lesions or in CL elicited by *L. major* a “wait and see” strategy can be practiced, ulcerative CL lesions leading to cosmetically relevant chronic wounds as well as lesions due to *L. tropica* infection usually require therapy. Local treatments recommended by WHO for the management of *L. tropica* infections include the intralesional injection of antimonials, the application of heat (thermotherapy, 50°C) or of liquid nitrogen (cryotherapy) [3]. In a randomized controlled trial (RCT) carried out in Kabul, thermotherapy using a radio-frequency generator was almost as effective as intralesionally administered sodium stibogluconate (SSG), leading to cure in 69.4% compared to 75.3% of the cases within 100 days after treatment initiation [15]. Other therapeutic strategies reported for *L. tropica*- or *L. major*-infected patients in Afghanistan include the use of oral hexadecylphosphocholin (miltefosine) or intravenous SSG [16], [17]. Overall, there is not only an urgent need for RCTs comparing the current therapeutic options for CL in the Old World [18], but also for the evaluation of novel treatments that are highly effective, well-tolerated, inexpensive and implementable in endemic countries [19].

A special pharmaceutical preparation of sodium chlorite (NaClO_2_) has been listed in the German Drug Codex as *sodium chlorosum* (DAC N-055) since 1990. It is produced in a chlorate-free manner by the reaction of chlorine dioxide (ClO_2_) with hydrogen peroxide (H_2_O_2_) under alkaline conditions and is saturated with carbonate. Besides the generation of NaClO_2_ the reaction of ClO_2_ and H_2_O_2_ presumably also leads to the formation of sodium salts of peroxychloric acid [20], [21]. In the presence of CO_2_/HCO_3_^−^ and ClO_2_^−^, peroxychloric acid is thought to stabilize as catalase-resistant peroxide salts (i.e. NaO[O = ]COOClO_2_ and Na_2_Cl_2_O_6_) [22], in analogy to the reactions described for peroxynitrite [23].

The pharmaceutical monograph of DAC N-055 is based on a 12 mM NaClO_2_ solution (pH 10 to 11) containing 2.4% glycerol, which in 1983 was approved by the German Health Authorities for the topical treatment of chronic wounds under the trade name of Oxoferin®. In 1989, the German Health Authorities banned the designation of tetrachlorodecaoxygen anions (TCDO; Cl_4_O_10_^n−^) as the active ingredient of Oxoferin®. A recent spectroscopic analysis of the water disinfectant HydroXan® [24], a compound similar to Oxoferin® (now Oxovasin®) and other commercial preparations of pharmaceutical NaClO_2_ (e.g., Ryoxon®, Oxilium®, WF10, or Immunokine™), confirmed that there is no evidence for the existence of the previously postulated TCDO complex consisting of chlorine (IV)-oxide and physically bound molecular oxygen [25]. Witke et al. [24] proposed a peroxide structure of ([ClO_2_]_2_O-O[ClO_2_]_2_)^2−^ for their “TCDO” preparation. However, this structure does not explain the strong chlorate-like Raman band they found and is therefore much less likely than the chlorine peroxide compound resulting from a chlorite stabilisation of peroxychlorate (e.g. ([Cl^III^O_2_]O-O[Cl^V^O_2_])^2−^) [22]. Under acidic conditions (pH≤6), in the presence of heme (Fe^3+^) [26] or manganese (Mn^3+^)-containing porphyrins [27], [28] or after exposure to ultraviolet A radiation the chlorite and/or the chlorine peroxide compound contained in DAC N-055 will release ClO_2_ and/or singlet oxygen (^1^O_2_), both of which are strong microbicidal oxidants [29], [30].

During the past 30 years *in vitro* analyses, animal studies and clinical trials have shown that preparations of pharmaceutical NaClO_2_ not only exerted antiviral and antimicrobial effects [31]–[34], but also promoted tissue regeneration and wound healing following radiation, chemotherapeutic, ischemic, metabolic, or infectious injuries [35]–[39]. Although the oxidative processes and molecular mechanisms underlying these findings have not yet been defined in detail, pharmaceutical NaClO_2_ clearly promotes cell proliferation, hematopoiesis, and microcirculatory blood and oxygen supply, modulates the functions of immune cells, and limits inflammatory responses [40]–[44].

Based on the hypothesis that ulcerative CL results from a defective wound healing response, the main goal of our study was to evaluate the effect of a careful wound management on the course and outcome of human CL. In the reported clinical trial carried out in Kabul, skin lesions of patients with CL were subjected to bipolar high-frequency electrocauterization (EC) followed by moist wound treatment (MWT) with or without DAC N-055. This new concept for the therapy of CL as well as preliminary results were first presented during an expert meeting at the Institute Pasteur, Paris, in 2006 [45].

## Materials and Methods

The original study protocol of this trial and the CONSORT checklist are available as supporting information (see **S1** Study Protocol and **S2** CONSORT Checklist).

### Objectives

The primary objective of this study was to evaluate the clinical efficacy of a new protocol for the treatment of *L. tropica* CL, which consisted of EC followed by MWT in the presence (group I) or absence (group II) of 0.045% DAC N-055 (which equals 5 mM pharmaceutical NaClO_2_). As secondary objectives we aimed to assess, whether continuous wound care might represent a valuable improvement of the therapy of CL compared to current anti-parasitic treatments and whether EC/MWT would positively influence the anti-leishmanial immune response and thereby helps to prevent relapses.

Our hypothesis was that EC plus MWT would lead to an acceleration of primary wound closure as compared to previous results obtained with the standard intralesional SSG injection. Furthermore, we hypothesized that EC plus MWT with DAC N-055 will be superior to EC plus MWT with physiological saline solution.

### Study site, design and participants

The study was performed as a two-armed, randomized, controlled, double-blinded, mono-centric clinical phase IIa trial at the Leishmania Clinic of the German Medical Service (GMS), Darwaze-e-Lahory, 1^st^ district, Kabul, Afghanistan, from 2004 to 2007.

Prior to the actual start of the trial, a pilot study at the GMS Leishmania clinic unexpectedly revealed that women and children, who were initially thought to be difficult to treat and follow for sociocultural reasons, were extremely reliable patients, who strictly observed their appointments and the medical instructions. On the other hand, the original assumption that a sufficiently high number of patients with non-ulcerated lesions can be recruited did not hold true. Therefore, in deviation from the original trial protocol, male *or* female patients of the GMS Clinic, 5 years of age or older, with non-ulcerated (papule/nodule) *or* ulcerated lesions (ulcer with or without crust) were eligible to the trial. Further inclusion criteria were the presence of at least one parasitologically confirmed CL lesion and no prior history of leishmaniasis and/or anti-parasitic treatment. Inhabitants of Kabul and its suburban areas were preferentially chosen in order to minimize bad compliance and drop-outs, as daily visits were needed during therapy. Exclusion criteria were the presence of CL lesion(s) on or immediately adjacent to the nose, lips or eyes; pregnancy; and any kind of serious comorbidities. In patients with more than one lesion, all lesions were subjected to the same randomly assigned treatment, but only one of them was selected as target lesion for the biopsies, the follow-up assessments and the final analysis.

### Ethics statement

The study protocol of the trial (ClinicalTrials.gov NCT00947362) was reviewed and provisionally approved on July 28, 2004, by the Ethics Committee of the University of Freiburg, Germany. Following two requested amendments, the full approval was issued on August 24, 2004 and submitted to the Afghan Ministry of Public Health in Kabul, Afghanistan on November 6, 2004. There were no Ethics Committees or International Review Panels according to Western standards at that time in Afghanistan. The study was conducted following the ethical principles provided by the Declaration of Helsinki.

As practically all patients could neither read nor write, an informed oral consent was obtained from them after detailed explanation of the study protocol by the medical doctor before the start of the treatment. All medical services and drugs provided during the trial were free of charge for the patients. The patients received no remuneration.

### Interventions

Prior to the treatment the medical history of each patient was taken and the present status of the lesion was recorded on a patient's card according to GMS standards (portrait photo of the patient, sex, age; comorbidities; site, size, Giemsa smear result and wound status of the lesion). To document the lesion and scar development, digital photographs were taken before, throughout and after therapy during each visit of the patients. The inclusion of a scale in the photograph allowed calculating the wound area by digital analysis with the help of the DatInf® Image DB software (DatInf GmbH, Tübingen, Germany).

After efficient cleaning of the skin with water and soap, the lesion site was disinfected with 70% ethanol and anesthetized with 1% lidocaine (AstraZeneca, Wedel, Germany) s.c. A 3 mm diameter punch biopsy (Stiefel Laboratorium GmbH, Offenbach, Germany) was taken from the edge of the lesion (1^st^ biopsy) and processed as described in the section *Parasitological Studies*. Superficial coagulation and removal of lesion tissue was performed using bipolar high-frequency electrocauterization (EC; also termed high-frequency electrocoagulation) with the help of the electrosurgical Mini Cutter HMC 80 HF (max. current in the bipolar mode 70 mA, KLS Martin, Umkirch, Germany) constructed with a specially designed bi-polar angled forceps with a 2 mm distance holder. Superficial coagulation of the epidermis was carried out with the strongest relative current (position 10) for approximately 2 sec. A constantly renewed thin layer of physiological saline above the skin lesion prevented tissue carbonisation. After EC, a polyacrylate hydrogel with or without freshly prepared 0.045% (w/w) pharmaceutical sodium chlorite (*sodium chlorosum*, DAC N-055, Waisenmedizin e.V., Freiburg, Germany; formula and preparation of the gel see supporting information **S3**) was applied to the lesions with the help of a sterile plastic syringe without a needle to avoid the rapid decomposition of DAC N-055 after transition metal contact. The wounds were dressed with sterile gauze and fixed with a bandage rather than plaster wherever possible. The MWT dressing was replaced daily with a weekly pause on Fridays at the Leishmania Clinic of the GMS until wound closure was achieved; if necessary, gentle wound cleaning was performed. In case of clinical signs for a superinfection, a smear was taken, Gram stained and microscopically evaluated for the presence of bacteria and/or fungi. Systemic antibiotics were only given, if bacteria were detected and the wound infection was severe. Neither local nor systemic antifungal therapy was allowed to exclude anti-protozoal effects on the *Leishmania* infection.

During the course of treatment Giemsa stainings of slit-skin smears were performed weekly. A 2^nd^ and 3^rd^ biopsy were taken after wound closure and 6 months later, respectively. The biopsies of the study patients were air-delivered with the help of diplomats and private passengers travelling from Kabul to Frankfurt/Main, Germany, and evaluated at the Department of Microbiology and Hygiene of the University Hospital of Freiburg or at the Microbiology Institute of the University Hospital Erlangen, Germany. The average transportation time was 4.06±1.90 days (mean ± SD).

### Outcomes

As primary outcome and end point for each patient within the study the time needed for complete epithelialization of the lesion wound was defined. The lesion status of all enrolled patients was routinely photo-documented when the MWT dressings were replaced and the number of healed wounds versus wounds that were still open was continuously recorded. As secondary outcome the parasite load within the lesion prior to the treatment start, at the time of wound closure and 6 months after wound healing was determined. In addition, adverse events such as bacterial or fungal superinfections of the wounds, the formation or scars and the rate of re-ulcerations were monitored during the treatment and follow-up period.

### Sample size

Sample size was calculated based on the primary outcome (i.e. complete wound healing). Considering the results of an earlier wound healing trial with Oxoferin® in patients with venous, arterial or postoperative ulcers [35], a mean healing time of four weeks in the control patients (group II) and of two weeks in the patients treated with DAC N-055 (group I) was assumed, with the maximal time for endpoint analysis being 60 days. Based on these assumptions a number of 40 patients each was calculated to be necessary to obtain a power of 80% (significance level 5%). A presumed drop-out rate of 20% led to a minimum total number of 100 patients to be enrolled. The prolonged wound healing times observed during the later phase of the study (year 2006 and 2007; see results) did not invalidate the sample size calculation, as the follow-up period was adjusted accordingly, i.e. all patients were followed up until wound closure.

### Randomization and blinding

In further specification of the original study protocol subjects were randomly assigned with the help of a GMS computer at a ratio of 1∶1 to group I (EC plus MWT with polyacrylate hydrogel including 0.045% DAC N-055) and group II (EC plus MWT with polyacrylate hydrogel without DAC N-055). The participants, the medical personnel treating and evaluating the patients, the investigators analyzing the clinical course of treated lesions, as well as the lab members performing the parasitological analyses were blinded. The preparation of the two different gels (marked as jelly A and B), which could not be visually distinguished, was performed by a technician who was not involved in the treatment of patients. RS and KWS were responsible for the enrollment of the patients and their assignment to the treatment groups.

### Parasitological studies

#### Immunohistology and limiting dilution analysis of skin biopsies

At the GMS Hospital in Kabul, *Leishmania* parasites within the lesions were detected using the slit-skin method [46] combined with Giemsa-staining and microscopic evaluation of the smears at 100× magnification. In addition, skin biopsies were taken from the margins of the lesions. Half of each biopsy was directly fixed in formalin for immunohistological analyses, the other part was placed into modified Schneider's *Drosophila* insect medium (Genaxxon BioScience, Ulm, Germany; supplements see [47]) to determine the parasite burden by limiting dilution (LD). For immunohistological analyses deparaffinized 3 to 4 µm tissue sections were treated with proteinase K solution (400 µg/ml, 5 min), H_2_O_2_ (0.3%, 15 min) and PBS containing 10% normal goat serum (Vector Laboratories, Peterborough, UK) and 5% bovine serum albumin (Roth, Karlsruhe, Germany) for 15 min. *L. tropica* parasites were detected by polyclonal rabbit anti-*L. major* serum [47] (dilution 1∶2000, 1 h) and visualized by biotinylated goat-anti-rabbit IgG (1 h), streptavidin-biotin peroxidase complex (30 min, both Vector Laboratories ELITE Kit ABC) and **3**,3′-diaminobenzidine substrate solution (Vector Laboratories). Finally, a counterstaining with hematoxylin (Sigma-Aldrich) was performed. For quantification of the parasite load within the skin biopsy by use of the LD technique, tissue homogenates were subjected to two-fold dilutions with 12 wells per dilution step and analyzed by Poisson statistics and the χ^2^ minimization method as described [47].

#### Determination of the Leishmania species

The *Leishmania* species was determined by mini-exon PCR and restriction fragment length polymorphism analysis as described [48]. Briefly, genomic DNA of the *Leishmania* parasites grown from the biopsies was extracted by a phenol-chloroform-based method, *Leishmania* mini-exon-specific PCR was performed, the resulting PCR fragment was individually digested by the restriction enzymes Eae I, Nco I and Hae III (New England Biolabs), and subsequently separated on a 4% agarose gel.

#### Cytotoxicity assay with extracellular Leishmania

To test the effect of DAC N-055 on extracellular parasites, *L. major* promastigotes (MHOM/IL/81/FE/BNI) [47] and the *L. tropica* isolates from the patients, which were propagated *in vitro* for a maximum of six passages, were seeded at 1000 parasites/100 µl per well in a 96-well plate and incubated for 4 to 7 days at different concentrations of DAC N-055 in modified Schneider's *Drosophila* insect medium. The growth of the parasites was recorded by measuring the optical density (OD) at 450 nm. After logarithmic transformation of the results, a sigmoidal curve fit and calculation of the mean with the 95% confidence interval and the half-maximal effective concentration (EC_50_) was carried out with Prism 4.0 software (Graph Pad, San Diego, California, USA).

#### Infection of human monocytes and intracellular parasite killing

Mononuclear cells from human peripheral EDTA-blood (PBMC) of healthy human volunteers resident in Erlangen and without any history of leishmaniasis were isolated using density centrifugation (1.077 g/ml Biocoll; Biochrom, Berlin, Germany). Monocytes were enriched either by depletion of CD3^+^CD19^+^ cells or positive selection of CD14^+^ cells applying MACS technology (Miltenyi, Bergisch-Gladbach, Germany).

The enriched monocytes were plated at 4×10^5^/400 µl in 8-well chambers slides (Lab-Tek Permanox, Thermo Scientific Nunc, Rochester, USA) and cultured for 20 h in RPMI1640 medium containing L-glutamin/NaHCO_3_ (Gibco, Life Technologies, Darmstadt, Germany) and supplemented with 50 µM 2-mercaptoethanol (Sigma-Aldrich), 10 mM HEPES (Gibco, Life Technologies), 100 U/ml penicillin G (Sigma-Aldrich), 100 µg/ml streptomycin (Sigma-Aldrich) and 5% (v/v) heat-inactivated (30 min at 56°C followed by sterile filtration with a 0.22 µm pore size filter) autologous human plasma. After three washing steps with PBS to remove non-adherent cells, adherent cells were infected for 16 to 19 h with stationary-phase grown *L. tropica* or *L. major* promastigotes (MOI = 5; 2×10^6^ *Leishmania*/400 µl), which had been opsonized by incubation in medium with 40% autologous human plasma for 5 min at room temperature. Thereafter, cells were again washed three times with PBS (time point 0 h) and cultured in medium or stimulated with different concentrations of DAC N-055 or with 20 ng/ml recombinant human IFN-γ (positive control for intracellular killing of the parasites, R&D Systems, Wiesbaden-Nordenstadt, Germany) for 48 h or 72 h. The infection rate and the parasite load per 100 infected adherent cells were determined microscopically after hematoxylin/eosin staining (DiffQuick; Labor-Technik, Berlin, Germany) of the cells.

### Statistical methods

All patient data were entered into Excel software (Microsoft) and statistically analyzed with SPSS (IBM® SPSS® Statistics version 20, SPSS Inc., Chicago, IL, USA) or GraphPad Prism version 4.0 (Graphpad Software Inc.). With respect to baseline and clinical data, the two randomized groups were compared using the Fisher's Exact test, the Mann Whitney test, the t-test or the Pearson Chi-Square test. The analysis of primary as well as secondary outcomes was planned on an intention-to treat basis (ITT) in this phase IIa trial. As we excluded those patients, who were lost during the follow-up period, the analysis of efficacy was *de facto* a per-protocol (PP) analysis. To compare the healing curves for the two treatment groups, the non-parametric log-rank test (Mantel-Cox test) was used; the Kaplan-Meier method was applied to evaluate the absolute wound closure time. The evaluation of differences in the parasite load between the two groups was performed with the help of a t-test. All *p* values are two-sided and *p*<0.05 was considered statistically significant.

## Results

A two-armed, randomized, double-blinded, monocentric clinical phase IIa trial was performed at the GMS Hospital in Kabul, Afghanistan, to evaluate the clinical efficacy of a new form of topical treatment of CL, which consisted of two key components, i.e. EC followed by daily MWT with polyacrylate hydrogel in the presence (group I) or absence (group II) of 0.045% pharmaceutical sodium chlorite DAC N-055.

### Participant flow and characteristics of the study population

**Figure 1:**
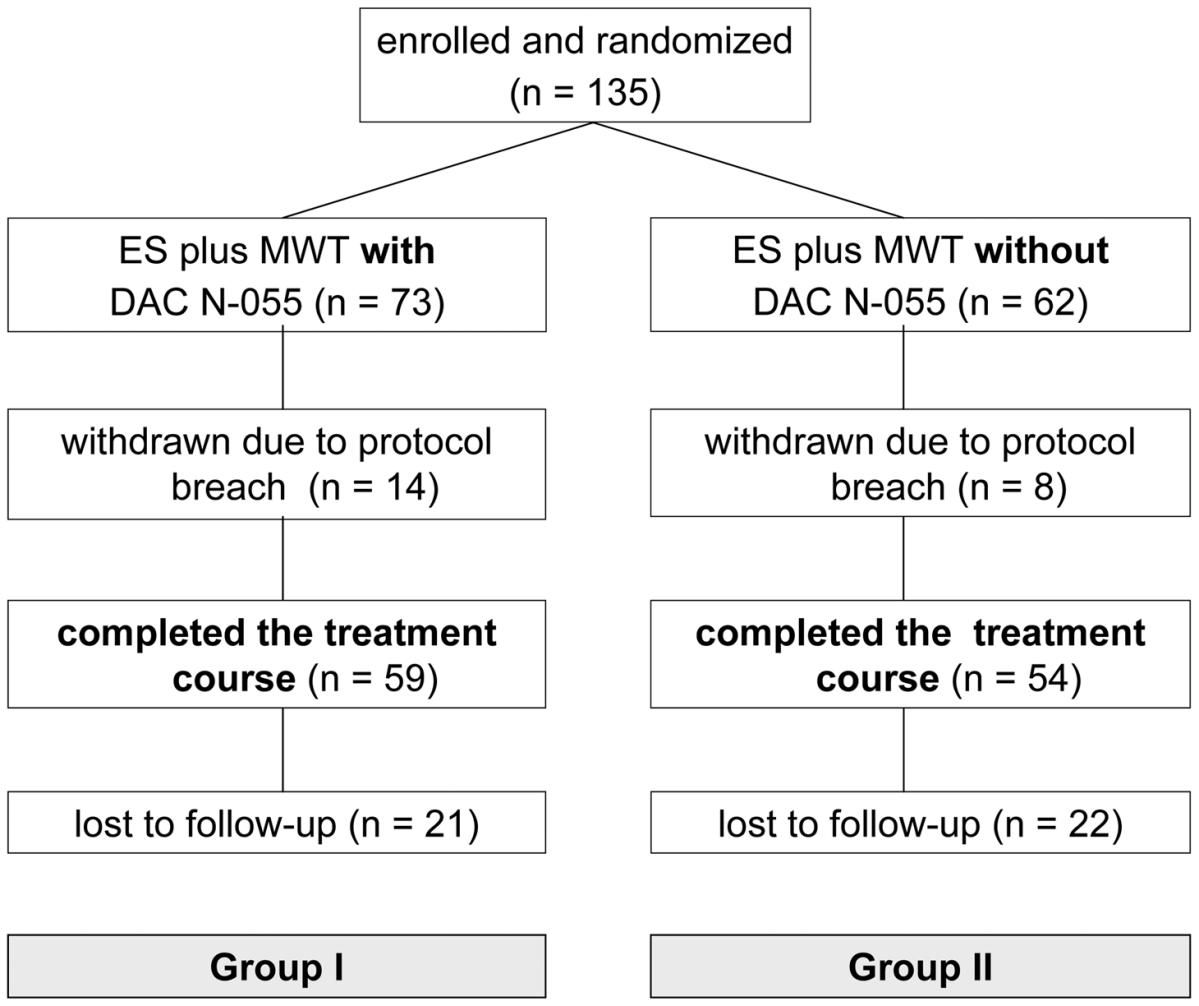
Study flow diagram showing the enrolment, randomization and follow-up of patients (CONSORT flow chart).

As detailed in **Fig. 1**, a total of 135 CL patients with at least one lesion, which was confirmed to be *Leishmania*-positive by Giemsa smear analysis, were included in the study. 73 of them were randomized to group I, 62 to group II. Out of the 135 patients, 14 patients of group I and 8 patients of group II were retrospectively excluded due to deviations from the study protocol (e.g. missing documentation, wrong wound treatment or interruption of the treatment by the patient). Thus, a total of 113 patients (59 in group I and 54 in group II) completed the assigned treatment and therefore were included in the PP analysis. For the follow-up analysis 6 months after wound healing, only 70 patients could be included, as 21 patients of group I and 22 patients of group II did not observe their appointments (**Fig. 1**).

*Leishmania* parasites were detected within all biopsies taken from the patients of group I and group II prior to treatment by (immuno)histology of formalin-fixed tissue (not shown). With the exception of a few samples secondarily contaminated by bacteria or fungi, all biopsies yielded the growth of *Leishmania* parasites. Genomic DNA was prepared from 58 parasite isolates, of which 28 were derived from group I and 30 from group II patients, covering the entire study period (11, 11, 21 or 15 isolates in the years 2004, 2005, 2006 or 2007, respectively). In each case *L. tropica* was identified as the underlying parasite species.

### Recruitment

Patients were enrolled from August 2004 until August 2007. The numbers of patients recruited per year were 19 (August–December 2004), 13 (January to December 2005), 29 (January to December 2006) and 52 (January to August 2007). The slow recruitment in 2005 and 2006 was due to a temporary lack of qualified personnel at the GMS.

### Baseline data

**Figure 2:**
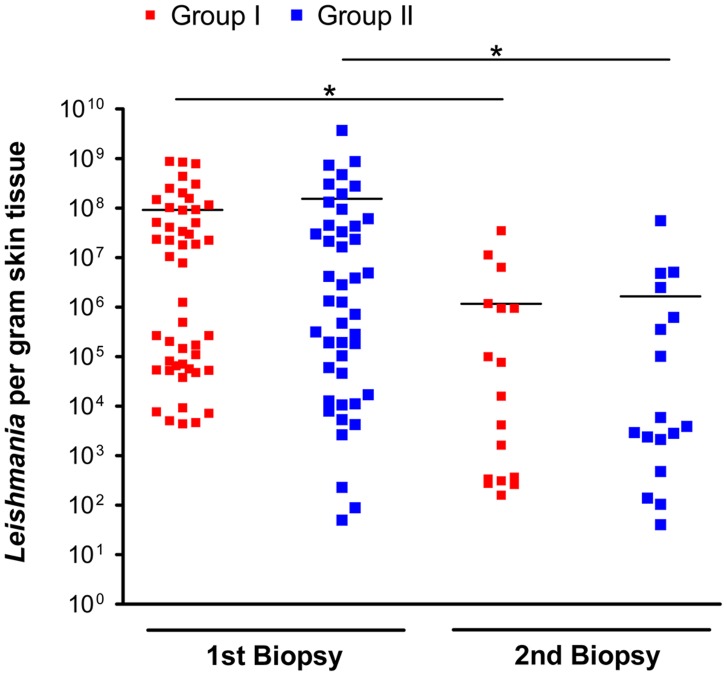
Parasite burden of the lesions before treatment (1^st^ biopsy) and after wound closure (2^nd^ biopsy). ▪ Patients, who received EC plus MWT with DAC N-055 (group I); ▪ patients, who received EC plus MWT without DAC N-055 (group II). Each symbol (▪, ▪) represents one biopsy; horizontal lines mark the mean value of each group. After wound closure (2^nd^ biopsy) 42 out of 59 biopsies in group I and 37 out of 54 in group II were *Leishmania*-negative (not shown). Level of significance was estimated using Student's t-test (*, *p*<0.05).

The baseline demographic characteristics, the localization and size of the lesion, the lesion type as well as the parasite load within the lesion before treatment start were comparable in the two treatment groups I and II (**Tab. 1** and **Fig. 2**). The quantity of *Leishmania* parasites per gram tissue in the 1^st^ biopsies was highly variable, ranging from 4.42×10^3^ to 8.75×10^8^ parasites/g in group I and from 8.9×10^1^ to 3.71×10^9^ parasites/g in group II, but was not significantly different between both treatment groups (**Tab. 1** and **Fig. 2**).

**Table 1:** Demographic, clinical and epidemiological profile of the study patients.

Variable	All patients	EC plus MWT with DAC N-055 Group I	EC plus MWT w/o DAC N-055 Group II	*p*
**Number of patients (ITT)**	135	73	62	
Sex				
Male	93	51	42	1.00^a^
Female	42	22	20	
Age (y)				
Mean	18.41	18.62	18.16	0.965^b^
Range	5–66	5–66	6–63	
Lesion type				
Papule or nodule	41	24	17	0.574^a^
Ulcer	94	49	45	
Lesion duration (m)				
Mean	2.77	2.92	2.61	0.402^b^
Range	1–12	1–12	1–9	
Localisation of the target lesion				
Face	36	22	14	0.310^c^
Shoulder/neck	2	2	0	
Upper extremity	86	42	44	
Lower extremity	11	7	4	
Giemsa smear				
G0 (no AM)	2	2	0	0.391^c^
G1 (1–10 AM)	89	49	40	
G2 (11–100 AM)	18	7	11	
G3 (>100 AM)	26	15	11	
**Number of patients (PP)**	113	59	54	0.359^a^
Parasite load in the 1^st^ biopsy (*Leishmania* per gram tissue): mean (SEM)	113	9.14 (2.85) ×10^7^	1.54 (0.83) ×10^8^	0.458^d^
Wound surface (mm^3^) after EC treatment: mean (SEM)	113	347.9 (23.39)	391.9 (27.80)	0,226^d^

Fisher's Exact test (^a^), Mann-Whitney test (^b^), Pearson Chi-Square test (^c^), t-test (^d^); m = months; y = years; AM = amastigotes; SEM = standard error of the mean.

### Efficacy of the treatment arms

#### Primary and secondary outcome and analysis of covariates

**Figure 3:**
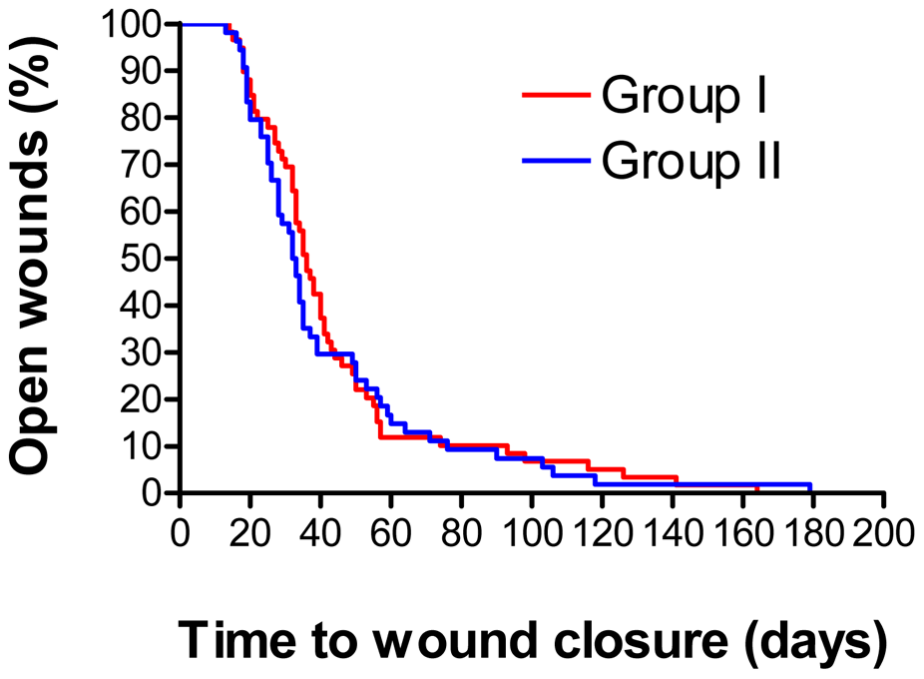
Wound healing in patients who received EC/MWT with (group I) or without DAC N-055 (group II). The data are presented in the format of a Kaplan-Meier analysis. No statistical difference was found in the log rank test Mantel Cox (*p* = 0.638).

The mean (± SD) time of wound closure for all patients enrolled in the PP analysis was 42.6 (±2.87) d after treatment start. The wound closure times in group I (EC+MWT with DAC N-055; 43.15±4.01 d) and group II (EC+MWT w/o DAC N-055; 42±4.13 d) were statistically not different (*p* = 0.83, *log rank* test Mantel-Cox). The Kaplan-Meier analysis based on the absolute days for wound closure revealed almost identical wound survival curves for treatment arm I and II (*p* = 0.0638, *log rank* test Mantel-Cox). 70% of all wounds were cured within 42 d in group I and within 39 d in group II. At day 74 to 76 after start of therapy, almost 90% (53 of 59 in group I; 48 of 54 in group II) of all wounds were completely re-epithelialized (**Fig. 3**). Depending on the year of enrollment, the mean wound closure times of all PP patients were 21.7 (±5.9) d (2004), 28.9 (±8.9) d (2005), 57.8 (±39.6) d (2006) or 48.0 (±34.0) d (2007). The increased wound closure times in 2006 and 2007 resulted from superfections, which occurred in both treatment groups during MWT (2006: 4 cases, 2007: 6 cases; see section on adverse reactions below). Superinfections clearly impaired the wound healing, as the mean (± SD) wound closure times of the PP lesions with or without superinfections were 132 (±34) d and 35 (±14.4) d, respectively.

**Figure 4:**
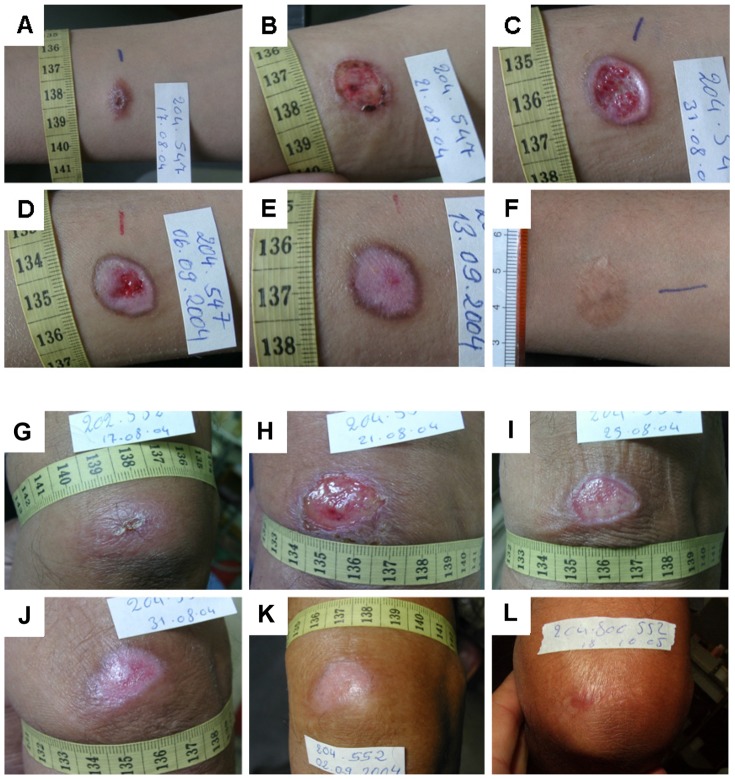
Representative examples of the tissue repair process and wound healing in the two treatment groups. **A–F**: 13 years old male CL patient treated with EC/MWT with DAC N-055. **A**: Ulcerated lesion before treatment located on the forearm. **B**: 4 d after EC, **C**: Formation of granulation tissue and beginning epithelialization after 15 d, **D**: Progressing epithelialization (d 19), **E**: Complete wound closure after 27 d, **F**: Follow-up after 20 months revealed a flat scar with hyperpigmentation. **G–L**: 63 years old male CL patient treated with EC/MWT without DAC N-055. **G**: Lesion on the upper arm with early ulceration prior to treatment. **H**: 4 d after EC, **I**: Formation of granulation tissue covered with fibrin and beginning epithelialization after 8 d, **J**: Progressing epithelialization (14 d), **K**: Complete wound closure after 16 d, **L**: Follow-up after 14 months revealed a flat scar.

As part of the medical observation of the patients during the treatment phase we continuously monitored the wound size. We observed no difference in the speed of wound surface decrease in the two treatment arms (data not shown). Representative examples of the photo-documented wound healing in both groups are given in **Fig. 4**.

Next, we performed a PP-analysis of a number of parameters (i.e. age, sex, wound size, parasite burden) that might influence the time to wound closure. Looking at the entire study population, the mean wound closure time of patients <18 y was 8.6 d longer than in the group of patients >18 y (45.4 d [CI 38–52.7] versus 36.8 d [CI 28.7–44.8], *p* = 0.06, *log rank* test Mantel-Cox) and the wounds of women tended to heal faster than those of men (38.7 d [CI 32.3–45.1] versus 44.3 d [CI 36.7–51.9], *p* = 0.63, *log rank* test Mantel-Cox). As the study population was equally distributed with respect to age and sex in group I and group II (**Tab. 1**), these baseline characteristics did not skew the results in the two treatment arms.

We also evaluated whether the size of the wound area resulting from the EC treatment influenced the healing. To this end, the entire study population was split into two groups with almost the same number of patients and a wound area larger or smaller than 317.6 mm^2^. Confirming earlier findings on chronic wounds of other origin [35], patients with a primary CL wound area after EC<317.6 mm^2^ cured roughly 10 days faster than patients with a wound area >317.6 mm^2^ (38.5 d [CI 29.9–47.0] versus 48.7 d [CI 40.4–56.9]). This difference, however, did not reach the level of significance (*p* = 0.06, *log rank* test Mantel-Cox).

**Figure 5:**
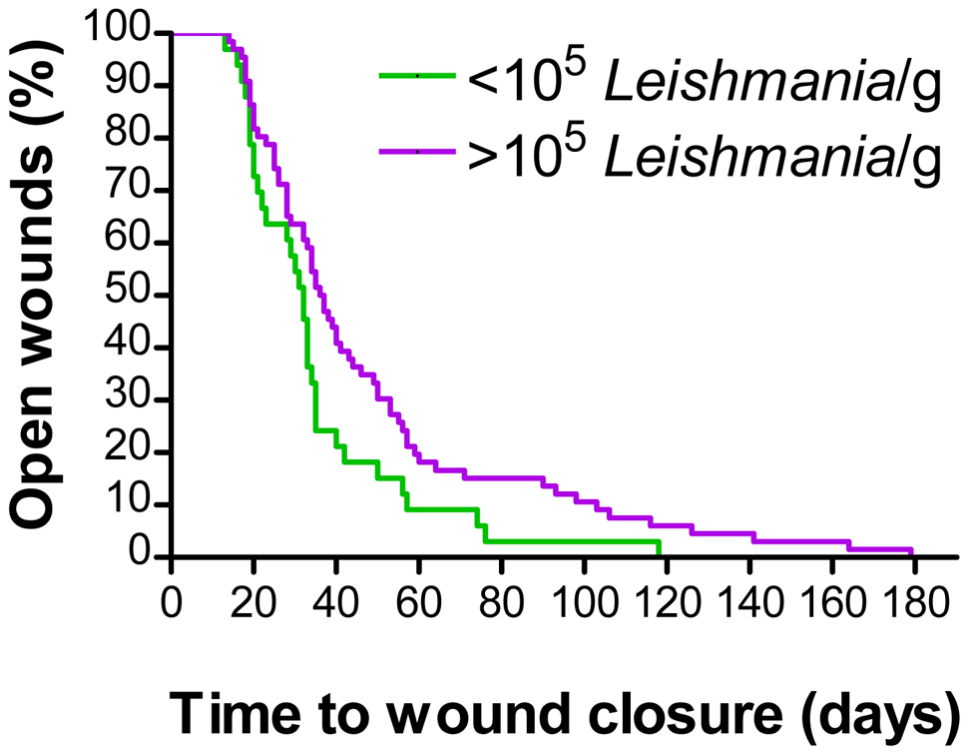
Impact of the lesional parasite load prior to treatment on the wound healing. The wound healing for patients of both treatment arms (PP analysis) with a parasite load higher of lower than 10^5^ per gram lesion tissue (1^st^ biopsy) was analysed using Kaplan-Meier curves. The log rank test Mantel-Cox revealed a significant difference (*p*<0.05).

With respect to the parasite burden in the skin lesion prior to treatment, we found that in patients with >10^5^ or <10^5^ *Leishmania*/g lesion tissue the average time to wound closure was 48.5 d (CI 27.8–41.4) or 35.1 (CI 39.7–57.2) days, respectively (*p* = 0.03, *log rank* test Mantel-Cox). This significant difference was also reflected by the Kaplan-Meier wound survival curves (**Fig. 5**). Both treatments ultimately led to an approximately 100-fold reduction of the mean parasite load (**Fig. 2**). In group I (n = 59), 17 patients showed *Leishmania*-positive 2^nd^ biopsies, whereas from 31 patients no more parasites could be cultured at the time of wound closure. Eleven 2^nd^ biopsies became contaminated with bacteria and/or fungi during or after the skin punch procedure; the resulting microbial overgrowth made a reliable assessment of the presence of parasites in the *Leishmania* cultures impossible. In group II (n = 54), there were 17 *Leishmania*-positive, 25 *Leishmania*-negative and 12 non-assessable 2^nd^ biopsies.

**Figure 6:**
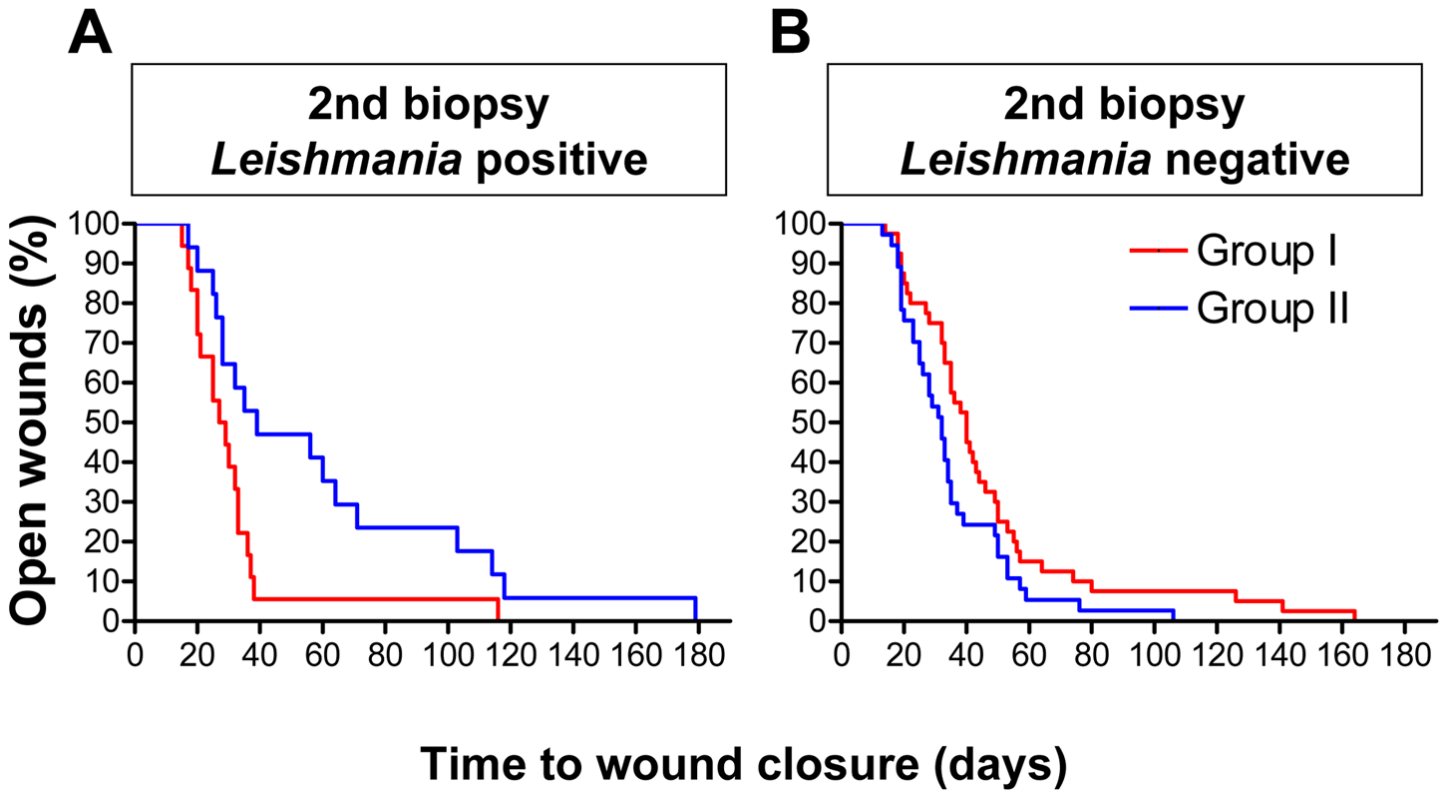
Kaplan-Meier analysis of the wound healing depending on the parasite status after treatment. The patients of both treatment arms (PP analysis) were grouped with respect to the presence or absence of *Leishmania* parasites in the 2^nd^ biopsy (i.e. after treatment). The wound healing was analysed using Kaplan-Meier curves. **A**: 2^nd^ biopsy *Leishmania*-positive (p = 0.08; log rank test Mantel-Cox). **B**: 2^nd^ biopsy *Leishmania*-negative (p = 0.2; log rank test Mantel-Cox).

As the amount of parasites persisting in the skin after wound closure was highly variable (**Fig. 2**), we also investigated whether there is a correlation of the wound closure times and the subsequent persistence of *Leishmania* parasites. In patients with persisting parasites (i.e. 2^nd^ biopsy was *Leishmania*-positive) the wounds epithelialized within 37.2 (+/−6.6) d when treated with MWT plus DAC N-055 after EC (group I), whereas the healing took 58.3 (+/−10.5) d, when patients received MWT without DAC N-055 (group II) (**Fig. 6A**). Compared to the patients with *Leishmania*-negative 2^nd^ biopsies (**Fig. 6B**), the differences between both treatment groups were considerably more pronounced, but still not significantly different (p = 0.08; log rank test Mantel-Cox) (**Fig. 6A**). Thus, we conclude that MWT in combination with DAC N-055 shows beneficial adjuvant effects particularly in patients with high and prolonged parasite burden.

#### Adverse events and reulcerations

Based on the clinical appearance of lesions following MWT and Gram-stains of microbiological swabs, bacterial and fungal superinfections occurred in both treatment groups at comparable rates (8.0% in group I versus 9.0% in group II; *p* = 1.000, Fisher's Exact Test). The total rate of superinfections within the entire study population was 8.8% in the PP-analysis. During the follow-up period, most of the patients in both treatment arms presented flat scars either with an erythematous central zone and hyperpigmented margins or with a completely hyperpigmented area, which faded away within months. In both groups of patients a similar incidence of keloïd formation was observed (5% in group I versus 6% in group II; *p* = 1.000, Fisher's Exact Test).

To further evaluate the efficacy of the two topical treatment strategies we investigated the rate of relapses. Altogether, 16 out of 113 patients (14.2%) developed reulcerations, which were insignificantly more frequent in group II (10 of 54 patients, 18.5%) than in group I (6 of 59 patients, 10.2%; *p* = 0.158, Fisher's Exact Test). A similar period of time was required in both groups to heal the lesions again (group I 69 d, group II 62 d). Except for one patient in group II, all 16 patients developing reulcerations were *Leishmania*-positive in the 2^nd^ skin biopsy (i.e. after primary wound closure). Together, these findings illustrate that leishmanial wounds can close in the presence of residual parasites. However, parasite persistence in the skin after wound healing facilitates the recurrence of the disease.

### Effects of DAC N-055 on extra- and intracellular *L. tropica* parasites

**Figure 7:**
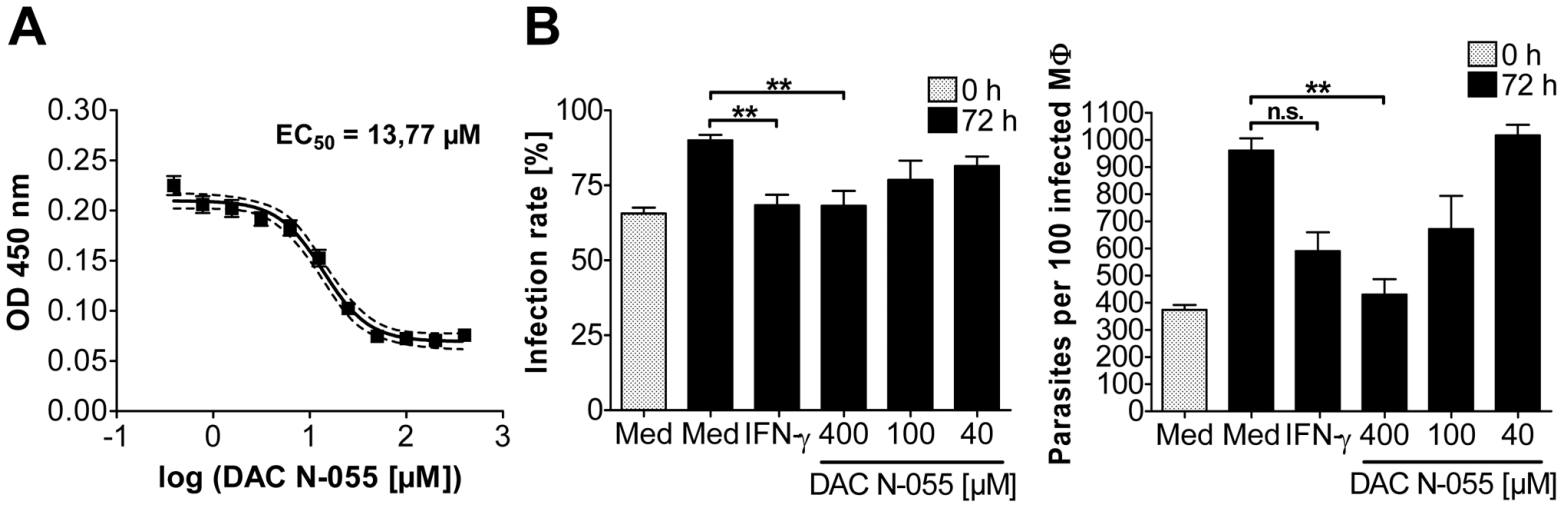
Effect of DAC N-055 on *L. tropica* parasites. **A**: Extracellular *L. tropica* promastigotes. The survival of extracellular *Leishmania* promastigotes after 4 d of incubation with DAC N-055 was determined via optical density at 450 nm. The OD values were logarithmically transformed and a curve fit was carried out. The mean of the curve (solid line) and the 95% confidence interval (dashed line) of two independent experiments with 24 OD values per DAC N-055 concentration as well as the ED_50_ are given. **B**: Intracellular *L. tropica* amastigotes. After infection of adherent human monocytes (macrophages, MΦ) for 19 h with stationary phase-grown and opsonized *L. tropica* promastigotes (MOI = 5) the cells were washed and stimulated for 72 h as indicated. The time point directly after the pulse infection was defined as 0 h. The infection rate and the number of parasites per 100 infected MΦ were determined at time point 0 h and after 72 h of stimulation. The mean ± SEM of three independent experiments are shown. For statistical analysis the Mann-Whitney test was used (** = *p*<0.001, n. s. = not significant).

The fact that 0.045% DAC N-055 (which equals 5 mM pharmaceutical NaClO_2_), showed beneficial effects especially in patients with high and persistent parasite loads, prompted us to investigate whether DAC N-055 has direct effects on extra- and/or intracellular *L. tropica* parasites. To this end, *L. tropica* promastigotes were cultured in the absence or presence of increasing concentrations of DAC N-055 (0.4–400 µM). The data revealed a clear cytotoxic effect of DAC N-055 on promastigotes, with a half maximal effective concentration (EC_50_) of 13.7 µM (**Fig. 7A**). Next, we analyzed whether this also holds true for intracellular *L. tropica*. Adherent human monocytes were infected with *L. tropica* promastigotes. Similar to the positive control IFN-γ, which is a known inducer of leishmanicidal activity in human mononuclear phagocytes [49], [50], DAC N-055 at concentrations of 40 to 400 µM that were non-toxic for the monocytes lowered the infection rate and blocked the replication of intracellular *L. tropica* parasites (**Fig. 7B**). Thus, the beneficial effect of DAC N-055 in human *L. tropica* CL might be partly related to its direct antileishmanial activity.

## Discussion

In the middle of the 20^th^ century it was first reported that the coverage of wounds with water-vapour permeable (i.e. semi-occlusive) synthetic foils generated a “moist chamber effect” [51] and promoted painless healing [51]–[54]. Today, semi-occlusive MWT is an established method for the therapy of different types of skin ulcers (e.g. diabetic foot ulcers, pressure ulcers or venous leg ulcerations) [55], [56], which has also been successfully used in countries of the Middle East [57]. In a recent meta-analysis of three RCTs [58] hydrogel as a wound moistener was superior to gauzes moistened with saline or iodine creme with respect to the closure of chronic wounds as final end-point. Hydrogels (e.g. polyacrylate) or hydrocolloids (carboxymethylcellulose) support the physiological wound autolysis and help to clean and debride necrotic and fibrotic tissue in the wound [56].

To our knowledge, the present study is the first which tested MWT in the context of a parasitic infection. We combined the MWT approach with the removal of lesional tissue by EC and obtained striking clinical effects. The observed wound healing times were considerably faster than those previously reported with the standard intralesional antimony treatment in the same endemic environment [15]. In the following we will discuss (a) possible mechanisms, (b) advantages, disadvantages and practical implications as compared to other therapeutic approaches, and (c) the limitations of our treatment protocol and study.

### Mechanism of action

The molecular mechanisms that account for the accelerated wound healing following MWT are only partially understood. First, early studies in pigs and humans provided convincing evidence that the epithelialization of covered, moist wounds progressed twice as fast as of wounds exposed to air. In wounds kept moist the newly formed epidermal cell layer migrated above rather than through the fibrous tissue deposited on the dermis; formation of scab and eschar was virtually absent [59], [60]. Second, loss of the integrity of the epidermis causes the immediate generation of an electric current in the skin [61]. Recently, it was shown that electric fields of a comparable order of magnitude directly alter the movement of epithelial cells *in vitro* via modulation of Src and inositol-phospholipid signaling pathways [62]. It is conceivable that MWT helps to maintain an electric gradient in the wound and thereby accelerates the migration of immune and epithelial cells. Finally, it has been proposed that MWT and occlusive dressings support the accumulation of wound fluid [63], which contains a cocktail of growth factors and cytokines essential for the repair processes [64].

The EC, which we performed prior to the MWT, resembles a surgical debridement practiced on chronic wounds with areas of necrosis and heavy slough. Presumably, it had two major effects: it caused a reduction of the parasite load, due to the coagulation and physical removal of tissue by heating the lesions with a high-frequency current; and it created a fresh wound bed for subsequent granulation and epithelialization. Unlike to the radio-frequency-induced heat therapy (50°C), which also proved effective in endemic areas such as Iran and Afghanistan [15], [19], [65], the sensitivity of *Leishmania* parasites to temperatures above 39°C is not the primary mechanism of action of our EC approach.

Considering the known tissue-regenerative activity of pharmaceutical NaClO_2_ [35]–[39], [65]–[67] and its cytotoxicity against extra- and intracellular *L. tropica* parasites (reported here), it was unexpected to see that the therapeutic benefit of including DAC N-055 in the EC/MWT protocol was confined to a subgroup of patients with a persistently high parasite loads in the 2^nd^ biopsy and to the partial prevention of relapses. A plausible explanation is that EC/MWT alone already exerted a strong anti-parasitic and wound healing effect, which in most patients could not be further enhanced by the addition of DAC N-055.

### Comparison with other treatment approaches

The current routine treatment for *L. tropica*-induced CL in Afghanistan consists of a course of intralesional or intramuscular injections of pentavalent antimony preparations (i.e. SSG or meglumine antimonate). To date, there is no RCT with a placebo group to directly support this standard therapy [19], [68], but clinical observations and two recent RCTs comparing antimony treatment and thermotherapy argue for the effectiveness of antimony in anthroponotic CL in Afghanistan [15], [65]. Nevertheless, there are several problems with the use of antimony-containing drugs. First, both the local and the systemic application can cause severe adverse reactions [69]. Second, the use of antimony has been linked to the emergence of drug-resistant parasites; antimony-resistant *L. tropica* strains and treatment failures have been reported from Iran [70]. Third, there have been recent incidences of limited supply and poor manufactural quality of antimony preparations in some countries [71], [72]. None of these concerns applies to our EC/MWT approach. The only possible side effects associated with MWT, especially if practiced with occlusive dressings, are superinfections and very rarely chloracne due to chlorine dioxide, which can be released from DAC N-055 under the acidic conditions of the skin. The incidence of superinfections, however, can be strongly reduced if a water-vapor permeable foil is used or the moist wound coverage is performed without foil as was the case in almost all of our patients. In the group of patients with MWT containing DAC N-055 no case of chloracne was observed.

A possible alternative to the high-frequency EC of the leishmanial skin lesions is the use of a CO_2_ laser [73], [74]. In Iran, the CO_2_ laser was more effective than systemic meglumine antimonate [75] or cryotherapy combined with intralesional meglumine antimonate in patients with CL [76]. However, for Afghanistan, one of the lowest developed countries in the world with an unstable electrical power supply even in the big cities and no infrastructure for technical maintenance, a CO_2_ laser is not the instrument of first choice for the treatment of CL lesions. Instead, the instrument for bipolar high-frequency EC used in this study is cheap and robust, needs no special maintenance, is suitable for small- and medium-sized CL ulcers and can be run with an easily rechargeable car battery.

Recently, two studies reported on the effect of wound care procedures in CL patients. In a three-arm clinical trial carried out in Iran with *L. major*-infected CL patients the additional administration of a triglyceride ointment dressing with or without silver impregnation failed to promote the healing of the lesions as compared to local injections of meglumine antimonate alone [77]. Although certain silver complexes might exert antileishmanial effects [78], [79], silver-containing dressings are no better or even worse than controls dressings in promoting wound healing [80]. In the second study, a highly heterogenous group of French travellers with CL (infections with 10 different species of *Leishmania* contracted in 29 different countries) either received simple wound care, cryotherapy plus intralesional antimony, or systemic drug therapy following the instructions by a reference center. Of 109 patients, only 25 patients were categorized to have simple lesions and received topical wound care, which led to cure in 23 patients. Unfortunately, no information was provided on the frequency and type of wound care performed [81].

In addition to pentavalent antimony other local or systemic drug treatments have been reported for *L. tropica*-induced CL. Both oral miltefosine and liposomal intravenous amphotericin B were effective in a few *L. tropica* CL cases reported [16], [82]–[84], but controlled trials are still missing. In addition, the current prices for these medications essentially prohibit their use in Afghanistan. The local treatment with paromomycin-based ointments, which have proven effective in ulcerative *L. major*-induced CL [85]–[87], did not yield convincing results in *L. tropica* infections (reviewed in [68]).

### Limitations

Due to the limited sample size of 59 and 54 patients we cannot firmly exclude the possible antagonistic influence of covariates (such as parasite load, age and gender), which might camouflage a significant difference between the placebo (EC/MWT without DAC N-055) and the verum group (EC/MWT plus DAC N-055) of the study [88]. Likewise, subsequent trials with a larger number of CL patients have to tell, whether non-desired effects of EC, such as keloïd formations and relapses of the L*eishmania* infection, are in the range of those observed with cryotherapy, thermotherapy, laser therapy, or local or systemic chemotherapy. Finally, the study design did not allow to judge the differential therapeutic contribution of the EC (reduction of parasite load by removal of lesion tissue) and of the MWT. In a follow-up three-arm study we therefore compared the therapeutic effect of EC plus MWT containing DAC N-055 with a wound-moistening treatment with DAC N-055 alone and with the standard intralesional injections of SSG (Stahl HC et al., in preparation).

In summary, the present study demonstrated that EC plus MWT is a straight-forward and clinically highly efficient method for treating CL lesions due *L. tropica* infections, which can be successfully implemented in a country such as Afghanistan, where infrastructural, financial and medical resources are limited. We strongly believe that MWT with DAC N-055 is a therapeutic approach that is likely to be also effective in patients with other forms of CL (e.g., infections with *L. major*, *L. mexicana* or *L. braziliensis*).

## Supporting Information

Supporting Information S1 Study protocol.
(DOC)

Supporting Information S2 CONSORT 2010 checklist.
(DOC)

Supporting Information S3 Formula of polyacrylate hydrogel with 0.045% *sodium chlorosum* (DAC N-055).
(DOCX)
