# Supplementary material for: Rapid Healing of Cutaneous Leishmaniasis by High-Frequency Electrocauterization and Hydrogel Wound Care with or without DAC N-055: A Randomized Controlled Phase IIa Trial in Kabul
Source: PLoS Negl Trop Dis. 2014 Feb 13;8(2):e2694. doi: 10.1371/journal.pntd.0002694 (PMC3923720; doi:10.1371/journal.pntd.0002694)
Supplement: Supporting Information S1 — Study protocol. (DOC) [file pntd.0002694.s001.doc]

**Protocol of the randomized double blind clinical trial** to be carried out in the GMS DL Clinic Kabul

**„Rationale for a new topical treatment of Afghans with Anthroponotic Cutaneous Leishmaniasis (ACL) with and without pharmaceutical chlorite listed in the German Drug Codex (DAC N-055)“**

Ahmed Fawad JEBRAN

9^th^ semester student of medicine at the Ruhr University of Bochum, Germany, Europe

Professor Dr. med. Christian BOGDAN

Chairman of the Department of Medical Microbiology and Hygiene, University Hospital of Freiburg, Medical Faculty of the University of Freiburg, Freiburg. Germany, Europe

Dr. rer. nat. Dr. med. Kurt-Wilhelm Stahl

Professor in an extracurricular capacity of Biochemistry of the Medical School of Hannover and Head of the NGO “Waisenmedizin (orphan medicine) e.V., Freiburg, Germany, Europe

Dr. Faquir Mohammad AMIN, MD, Dipl. Epidemiologist,

Medical Director of the DARWAZE-E LAHORY (DL) Clinic

Reto STEINER

GERMAN MEDICAL SERVICE (GMS)

Administrator of the DL Clinic


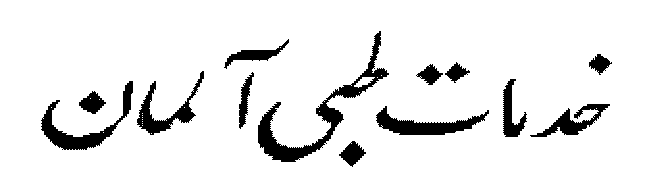


**Kabul, Post Office Box**

**Mobile Phone 070 277 209 / 210**

**Sat-Tel: 00873-762930465 / Sat-Fax: 00873-76293066 /
e-mail: CT.Kabul@t-online.de Registered I. NGO No. 35**

**Kabul, Afghanistan 12-06-2004**

submitted to the

**Ethics Commission of the University of Freiburg, Freiburg, Germany (No. 169/04)**

& to the

**Ministry of Health (MoH) of the Transitional Islamic State of Afghanistan in Kabul**

**Background**

The intralesional and systemic treatment of active **anthroponotic cutaneous leishmaniasis** (ACL) with pentavalent antimonials (Sb^V^), which are in use since 1929, are still the current main therapeutic strategy in Afghanistan. The intralesional approach is unsatisfactory because of pain and negative toxic effects on wound healing. In approximately 10 % of the cases both systemic as well as intralesional administrations require discontinuation because of allergy. Other serious side-effects are myalgia, pancreatitis, renal failure, peripheral neuropathy, hepato- and cardiotoxicity.

In contrast to the systemic and mucocutaneous forms of leishmaniasis, which are predominant on the Indian subcontinent and in South America respectively, ACL, the overwhelming form of *Leishmania* disease in Afghanistan, is a self-limiting parasitic infection. In patients in Afghanistan, 2 to 24 months after the transmission of *Leishmania (L.) tropica* by the sand fly (*Phlebotomus sergenti*) (incubation period), a shiny papule slowly growing to a nodule is formed, which ulcerates centrally, enlarges centrifugally and has the tendency to heal after one to two years without any treatment leading to a permanent immunity in ca. 90 % of the cases. In 10% of the cases relapses with scant parasites in the lesion, an increased delayed type hypersensitivity (DTH) reaction to parasite antigens and/or resistance to SB^V^ treatment occur. *L. tropica* rarely spreads to the viscera.

However, terrible **hygienic conditions** in the extremely dry and dusty climate of Afghanistan constitute serious problems for any long-lasting wound in this country. Actually, Kabul is without any sewage system for its 3.5 million inhabitants. Here, chronic ulcers rapidly cover with thick crusts which are super-infected with fungi and bacteria and lead to a high incidence of chronic eczemas and to disfiguring scars that result in social exclusion of girls and women, if they are located in the face.

The wound situation of ACL in Afghanistan is worsened by an over-treatment of many patients with intralesionally administered Sb^V^ drugs, because this pays well off for doctors making “joint ventures” with pharmacists: Drugs are either sold at excessive prices (e.g. a vial with 1.5 g Glucantine for 1.5 US $) or they are sold after improper storage (e.g. K.-W. S. has seen patients bringing Glucantime from the pharmacy showing a yellow colour).

The **ACL incidence** is high in endemic regions of Afghanistan. Kabul is currently witnessing the world-wide biggest cutaneous leishmaniasis epidemic with actual estimates ranging between 67.500 and 200.000 patients (“A randomized controlled trial to test the efficacy of thermotherapy against *L. tropica*”. Final report 2004, Health*Net* International, Kabul).

In autumn 2002, the moist facial ACL ulcer of a 7 year old boy, which had persisted for 7 months and had meanwhile turned Leishmania-negative in the Giemsa stained wound smear, healed within days, when the wound surface showing a diameter of 4 cm was treated with a 0.045% pharmaceutical chlorite solution in physiological saline at the DL clinic in Kabul (German Medical Service [GMS], case report to be submitted for publication).

This result prompted the DARWAZE-E LAHORY clinic of the GMS in Kabul, which has treated 1.223 ACL cases in 2003, to approach the ACL treatment as a wound care management rather than as an anti-parasitic therapy.

**Pharmaceutical sodium chlorite** entered the German Drug Codex in 1990 under DAC N-055, i.e. 7 years after a pharmaceutical sodium chlorite preparation had been approved for moist wound care management by the German Health Authorities, the former BGA in Berlin, under the trade name of OXOFERIN®. Originally, it was believed that technical grade sodium chlorite purchasable as a chemical compound bulk ware could do the “drug job” of OXOFERIN®. However, only recently it became clear that chlorite which had been produced from chlorine dioxide in a chlorate free manner by the addition of peroxide under alkaline conditions, lost its strikingly positive clinical effects on wound healing, which have been described in several publications for the registered finished chlorite solution OXOFERIN® , after it had been either concentrated by evaporation or purified through crystallisation. It is currently believed that alkaline chlorate-free chlorite production with chlorine dioxide as starting material leads to the formation of the salt of peroxychloric acid , which yields pure chlorite and oxygen when evaporated to dryness.

Thanks to financial support in Germany by Caritas International, Diakonie – Katastrophenhilfe, and BASF AG Ludwigshafen, the non-profit non-governmental organisation (NGO) “Waisenmedizin (WM e. V.)” in Freiburg, was able to produce pharmaceutical chlorite which is currently not available on the German drug market, first in 2002 and then in 2003. The 4.5 g% (m/m) drug concentrate is donated to Afghan institutions by WM e. V.

For ACL ulcers with crusts, the GMS meanwhile uses a polyacrylate hydrogel with 0.045% pharmaceutical chlorite (DAC N-055), which the WM e. V. member and pharmacist, Dr. Kurt Bauer, Emeritus Professor of the Pharmaceutical Faculty in Freiburg, proposed in slight modification of a DAB formulation.

Pharmaceutical sodium chlorite hydrogel has been used meanwhile by the GMS in Kabul to promote healing of more than 80 chronic ACL ulcers with dry crusts.

As an alternative to the Sb^V^ treatment, the GMS started to treat fresh ACL nodules with **electrocauterisation (EC)** on a pilot basis since the beginning of 2003, as it was believed that such heat treatment could reduce the number of parasites allowing the immune system to cope with the remaining protozoa in a more efficient manner. Limiting dilution analyses carried out at the Department of Medical Microbiology and Hygiene, University Hospital of Freiburg, showed that the *Leishmania* parasites were still present in the tissue when the punch biopsy was taken under local anaesthesia shortly after EC before starting moist wound treatment (MWT).

Interestingly in 2004, the Dutch NGO in Kabul, HEALTH*Net* International, which cooperates with the London School of Hygiene & Tropical Medicine, filed a final report on a randomised controlled trial to test the efficacy of thermotherapy (TT) at 50 °C (TT without cauterisation) against *Leishmania tropica* versus intralesional and systemic Sb^V^ therapy. Based on Giemsa smears with PCR confirmation of Leishmania etiology in 10% of the enrolled patients, they found that TT (69.4 % cure rate after 3 months, mean duration to cure 43.46 days) was as effective as intralesional Sb^V^ (75.3 % cure rate after 3 months, mean duration to cure 58.9 days) and more effective than i. m. Sb^V^ (44.8% cure rate after 3 months, mean duration to cure 49.46 days). They enrolled 406 patients, but their total drop out rate during a follow-up period of 3 months was 36.2 % in Kabul.

When the GMS subsequently treated the EC wounds of their patients with 0.045% DAC N-055 containing hydrogel, this led to a complete epithelialization of the majority of lesions with depigmentation and edge flattening mostly within 4 weeks. Giemsa smears, which were found to be *Leishmania*-positive before EC, turned *Leishmania*-negative within 7 days and stayed negative until complete healing. The punch biopsies of two of such lesions were transported from Kabul to Freiburg within 48 hours in *Leishmania* growth medium, where they were subsequently found *Leishmania*-negative in limiting dilution analyses. However, in chronic ulcers which had not been pre-treated by EC, in some cases a discrepancy was found between Giemsa smears which had turned *Leishmania*-negative and limiting dilution analyses which remained *Leishmania*-positive.

**Methods**

**Study location**

The clinical study will be carried out at the GERMAN MEDICAL SERVICE (GMS), Darwaze-e Lahory (DL) clinic, in Kabul, Afghanistan. The clinic has been operating for more than two decades. Being mainly involved in leprosy and tuberculosis programs, the DL clinic also focuses on dermatological patients. 23.5% of all patients seen in 2003 presented with cutaneous leishmaniasis. Recently, the DL clinic has accumulated a lot of experience in moist wound care management with dressings and digital photo documentation of as many as 50 patients per day.

**Study design**

1. The study will be a **randomized controlled double-blind trial**. Neither of the two groups is a placebo group, which would be unethical considering the dermatological virulence of *L. tropica*, because EC and moist wound treatment (MWT) have been seen to exert a therapeutic effect (GMS, unpublished results) similar to thermotherapy (“A randomized controlled trial to test the efficacy of thermotherapy against *L. tropica*”. Final report 2004, Health*Net* International, Kabul).

Since early 2003, electrocauterisation (EC) has become a routine therapeutic alternative to Sb^V^ injections for ACL patients at the GMS DL clinic. If initial EC is combined with subsequent MWT, this leads to cosmetically more acceptable healing results in a shorter time. It promotes granulation and in the ongoing open prospective pilot trial MWT promises to be more effective (3 relapses out 87 cured cases) than subsequent dry wound care with disinfectants (3 relapses out of 24 cured cases).

To elucidate the drug factor in MWT, a randomized double blind trial comparing electrocauterisation (EC) followed by moist wound treatment with or without 0.045% DAC N-055 in sodium polyacrylate hydrogel is to be undertaken now in patients with ACL lesions.

1. **Study patients, inclusion and exclusion criteria**

Eligible are patients attending the GMS DL clinic, who present at least one suspected ACL lesion on their arms or legs with no other skin diseases or infections and who have never been previously treated by Sb^V^ injections or any other form of anti-leishmanial medication. The lesions must be in the non-ulcerated stage.

Other inclusion criteria are a minimum age of 18 years, male sex and a laboratory confirmation of ACL by Giemsa stained wound smears before treatment.

Female patients and children are excluded from the trial to limit drop-outs due to the fact that for sociocultural reasons the treatment of women in Afghanistan might be too complicated as they normally require a male escort and that the skin lesion biopsies might deter children and females from returning for the follow-up appointments. Further exclusion criteria are the presence of ACL lesion(s) on or immediately adjacent to the nose, lips or eyes and any kind of serious comorbidities.

1. **Sample Size**

In view of potential drop outs, 2 x 50 eligible patients are to be enrolled into this study within two weeks.

**Objectives**

The aim of the trial is to

- evaluate the clinical efficacy of two new topical treatment forms of ACL: electrocauterisation (EC) followed by moist wound treatment versus EC followed by moist wound treatment plus 0.045% pharmaceutical chlorite that has been used in three European countries (Germany, Austria and Switzerland) in wound care management for over 20 years
- judge whether early wound care management would present a viable improvement to the actual anti-parasitic treatments mostly neglecting the chronic wound problem and to evaluate its long-term effect on immunity through relapse control 6 months after wound healing.

**Interventions**

1. **Skin biopsies**

Two punch biopsies are taken from every patient under local anaesthesia, the first before EC, the second after complete wound healing. One half of the 2.0 mm diameter cylinder is transferred into tubes with *Leishmania* culture medium under sterile conditions for limiting dilution analysis (LDA) within 48 hours to the Department of Medical Microbiology and Hygiene of the University Hospital of Freiburg. The second half is fixed in formalin for histopathology in Freiburg. For their second punch biopsy the patients will be examined in clusters. The second punch biopsy should be done within one week after complete healing to avoid drop outs. From wounds which have not completely healed after 8 weeks of MWT, a second biopsy will also be taken the following day.

1. **Electrocauterisation (EC)**

After intensive skin cleaning with water / soap first followed by rinsing with 70% ethanol and disinfection of the intact skin in both groups **A** and **B** with a 5% dilution of 4.5% (w/w) pharmaceutical chlorite plus 1.5 vol% acetic acid for 20 minutes, the nodule is locally anesthetized with 1% lidocaine. The whole nodule area is then electrocautered with a high-frequency electrosurgical Mini Cutter (HMC 80 HF Chirurgie) produced by Hüttinger Medizintechnik (D-79224 Umkirch) first using monopolar superficial coagulation and then monopolar deep coagulation for thermo-therapy starting with intensities from 4 to 6 depending on the thickness of the epidermis and using a broad blade and intensities between 5 and 7 after the skin has been opened.

**C. Moist wound treatment and wound dressings**

0.5 ml of 4.5% (w/w) pharmaceutical chlorite solution per 50 g jelly (which equals a concentration of 5 mM NaClO_2_) pH 7 and drug free jelly pH 7 are freshly prepared in the GMS laboratory every day according to the attached “mucilago” formulation. The pots with the hydrogels are marked jelly A and B without any further indication for the personnel doing the wound dressing. The jellies are applied directly to the wounds with a sterile syringe, which are then covered with gauze and closed with a layer of food preservative folia fixed with a bandage, after the whole skin area covered by folia has been intensively cleaned with 70% ethanol. Dressings are renewed every day with a weekly pause on Fridays. The wounds are checked for possible superinfections by Giemsa-stained wound smears. If dry gauze dressing for one day does not change the situation, systemic antibacterial treatment is given. Anti-leishmanial and topical co-medication is not allowed. If a patient is over-due for his daily dressings for more than two days, he/she is excluded from the trial.

**Outcomes and efficacy endpoints**

Both forms of treatment, EC plus polyacrylate dressing with or without pharmaceutical chlorite DAC N-055, are expected to be beneficial for the patient. However the reactive oxygen intermediate carrier, pharmaceutical sodium chlorite, which has been proven to enhance tissue regeneration and to exert antimicrobial effects, could also have a beneficial effect against the *Leishmania* parasite invader. This is to be checked as follows:

The time of complete wound healing is registered for each wound. After a maximum of 60 days of MWT the number of all healed wounds (complete epithelialization documented by photo) is registered versus the number of all wounds still open at that time. The parasite load is determined by limiting dilution analysis (see below) in a 2^nd^ skin biopsy taken after complete healing or on day 61 after EC, if the wound has not healed completely.

GMS plans a late control of the ACL patients after six months with photodocumentation, Giemsa smear and eventually a 3^rd^ biopsy for LDA, if there is still a tissue infiltration but the Giemsa smear is Leishmania-negative and if the patient is willing to consent to this.

**Laboratory and parasitological studies**

1. **Wound smears** are taken weekly from the wound edges and analysed in the GMS laboratory to follow the development of the parasitological disease: The slides are dried, fixed with methanol, stained with Giemsa and then examined under the microscope at x100 magnitude for presence of the *Leishmania* amastigotes.
2. **Limiting dilution analyses** **(LDA)** are done to determine the parasite load per g of biopsy tissue in the Department of Medical Microbiology and Hygiene of the University Hospital of Freiburg, Germany, according to the method established in the laboratory of Professor Dr. Christian Bogdan. It is intended that the biopsies are subjected to analysis within 2-4 days after biopsy transfer into *Leishmania* medium avoiding exposure to temperatures above 25°C. For LDA, the biopsy sample will be minced and subjected to serial 2-fold dilutions (total number of dilutions = 15 ranging from undiluted to 1:32,768, number of wells per dilution step = 12) in modified Schneider´s Drosophila insect medium . The cultures are kept for a minimum period of 10 days, after which they are evaluated microscopically for the growth of *Leishmania* promastigotes. The number of parasites is estimated using Poisson statistics and the χ2 minimization method .
3. **The determination of the parasite species** is done by PCR and restriction fragment length polymorphism analysis .

**Statistical analysis**

Statistical tests will be carried out according to standard methods as advised by the Department of Medical Biometry and Statistics, University of Freiburg.

**Cited literature**

Freiburg, Kabul, 12^th^ of June 2004

Dr. Faquir Mohammad Amin, MD, Dipl. Epidemiologist,

Medical Director of the GMS DL Clinic

Reto Steiner, Administrator of the GMS DL Clinic

Professor Dr. rer. nat. Dr. med. Kurt-Wilhelm STAHL, presently in Kabul

Head of the NGO Waisenmedizin e.V. Freiburg Germany

Professor Dr. med. Christian Bogdan, Head of the Department of Medical Microbiology and Hygiene at the University Hospital of Freiburg

**Amendments to the original study protocol from June 12, 2004**

Amendment 1 (requested by the Ethics Committee of the University of Freiburg on July 28, 2004; submitted August 11, 2004; approved August 24, 2004): Informed consent of the patients

The patients will be informed orally by the doctors or the medical student about the planned diagnostic and therapeutic procedures. Specifically, they will hear about the advantages (e.g. more rapid cure, less infections, healing of the skin plus killing of the parasites) as well as the possible side effects of the new treatment (e.g. allergic reaction to the foil or the pharmaceutical chlorite) as compared to the established standard methods.

Amendment 2 (requested by the Ethics Committee of the University of Freiburg on July 28, 2004; submitted August 11, 2004; approved August 24, 2004):

Sample size and power calculation, randomization, primary and secondary endpoints

For this study, 100 patients shall be recruited, 50 in each treatment arm, eventually for technical feasibility reasons in a sequential order, 2 x 25 patients during the first 2 months and 2 x 25 patients during the following 2 months.

*Randomization* will be performed in two strata: patients with a single lesion and those with multiple lesions. In the latter case, one target lesion will be determined in advance for the primary evaluation; other lesions will be documented for further investigations.

*Primary endpoint* is the time until complete wound healing. Under the assumption that the median healing time is 4 weeks in the control arm and 2 weeks in the investigational arm and that the follow-up time is 60 days, a number of 40 patients each is necessary to obtain a power of 80% (significance level 5%). A presumed drop-out rate of 20% leads to the total number of 100 patients. Comparison of the study arms will be performed in the ITT population by means of the Log-rank test. Depending on the data, a more detailed analysis in a Cox Model including single/multiple lesions and parasite load at baseline as additional effects might be performed.

As a *secondary endpoint*, parasite load after 60 days will be compared in the PP population between the two study arms either by means of the Mann-Whitney-Wilcoxon test or in case that the distribution shows a singularity at zero, by means of the fourfold table chi-square test (dichotomized neg/pos).
