# Supplementary material for: Rapid Healing of Cutaneous Leishmaniasis by High-Frequency Electrocauterization and Hydrogel Wound Care with or without DAC N-055: A Randomized Controlled Phase IIa Trial in Kabul
Source: PLoS Negl Trop Dis. 2014 Feb 13;8(2):e2694. doi: 10.1371/journal.pntd.0002694 (PMC3923720; doi:10.1371/journal.pntd.0002694)
Supplement: Supporting Information S3 — Formula of polyacrylate hydrogel with 0.045% sodium chlorosum (DAC N-055). (DOCX) [file pntd.0002694.s003.docx]

**Supporting Information S3:** Formula of polyacrylate hydrogel with 0.045% *sodium chlorosum* (DAC N-055)

| **Compound** | **Gram (g)** |
| --- | --- |
| Polyacrylic acid (Carbomer 50.000) | 0.25 |
| Glycerol | 13.0 |
| Sterile distilled water | 34.50 |
| Sodium hydroxide solution 5% | 1.75 |
| Sodium chlorite solution (*sodium chlorosum*, DAC N-055) 4.5% (w/w) | 0.50 |
| Total amount of hydrogel (adjusted to pH7-8 with NaOH, pH stick controlled) | 50 |
